# Dissecting the Role of Reduced Graphene Oxide on the Dielectric Properties of Poly(vinyl alcohol)/Poly(3,4-ethylenedioxythiophene):Poly(styrenesulfonate) Blend

**DOI:** 10.3390/polym18192403

**Published:** 2026-10-01

**Authors:** Mydhili Varma, Sellaperumal Manivannan, Paul Ben Ishai

**Affiliations:** 1 The Dielectric & THz Science Laboratory, Department of Physics, Ariel University of Samaria, Ariel 4070000, Israel; 2 Carbon Nanomaterials Laboratory, Department of Physics, National Institute of Technology, Tiruchirappalli 620 015, India

**Keywords:** dielectric relaxation, PVA, reduced graphene oxide (RGO), PEDOT:PSS

## Abstract

Herein, we present the fabrication of freestanding films of a reduced graphene oxide–poly(3,4-ethylenedioxythiophene):poly(styrenesulfonate)–poly(vinyl alcohol) (RGO–PEDOT:PSS–PVA) composite through a solution blending method at optimal synthesis conditions and varied RGO concentration. The frequency- and temperature-dependent dielectric properties were measured within the temperature range of 303–523 K for the frequency range 10^2^–10^6^ Hz. The temperature-dependent dielectric spectra suggest that, at the low-temperature regime (<358 K), polarization occurs due to the water molecules or -OH groups present in the composite, which is supported by the thermogravimetric analysis of the sample. Above 358 K and 100 Hz, the steep rise in the dielectric constant (ε′) and dielectric loss (ε″) to the order of 10^5^ and 10^6^, respectively, suggests an increase in the dc conductivity due to percolation of charge carriers. The RGO acts as a channel for charge percolation and transport and forms a barrier between the PVA and PEDOT:PSS through intermolecular hydrogen bonding and π−π interaction between the thiophene ring in PEDOT:PSS and sp^2^ carbon of RGO. The FTIR and microRaman data supports our claim along with dc conductivity data obtained from the Havriliak–Negami model. The temperature-dependent dielectric studies suggest the role of RGO in the polarization and relaxation mechanism of the composite and give an idea about the range of temperature at which the sample can be effectively used as a dielectric for practical use.

## 1. Introduction

The rational design of high-performance dielectric materials is one of the major foci in the fields of advanced energy storage systems, high-*k* gate dielectrics, electromagnetic interference shielding, and electroactive materials [1,2]. Among the high-performance dielectric materials, polymer-based dielectric composites are favorable due to their high dielectric breakdown strength, improved mechanical strength, excellent electrical insulating performance, light weight, low cost and easy processing for large area application [3]. This can be achieved through the strategic incorporation of nanofillers of various dimensions, such as 0D nanoparticles, 1D nanowires/nanotubes/nanofibers and 2D nanosheets/nanoflakes/nanoplates, into the polymer matrix. This influences the crystalline behavior and the mobility of the amorphous components of the polymer matrix [4]. The commonly used fillers are inorganic or carbon-based or organic fillers. Some commonly used organic fillers are crosslinked polypropylene (CPP), conducting polymers like PEDOT and its derivatives, PPy, PANI, fluorinated molecules, and PTFE coatings [5,6,7,8,9,10]. A few commonly used inorganic fillers are ceramics, metal nanoparticles, carbon nanomaterials, and metal oxides or their combinations, such as K_2_Ni_0.93_Ti_7.07_O_16_ (KNTO) with Ag coating as the nanofiller, Na_1/3_Ca_1/3_Bi_1/3_Cu_3_Ti_4_O_12_ (NCBCTO) ceramic particles, core–shell structure hybrid powder of SrTiO_3_@MWCNTs, (La_0.5_Nb_0.5_)_x_Ti_1-x_O_2_ ceramic nanorods (x-LNTO NRs) and three-dimensional fiber structure network (3D-1-LNTO), Al_2_O_3_, core-shell BaTiO_3_@SiO_2,_ MXenes, ZnO@MoS_2_, etc. [11,12,13,14,15,16,17,18]. Among the various polymer matrices, like PVDF, PEEU, and BOPP, poly(vinyl alcohol) (PVA) has established itself as an ideal platform for investigating fundamental structure–property relationships in dielectric composites due the semicrystalline morphology of PVA chains with abundant hydroxyl groups that provide better filler dispersion and interfacial interactions. However, PVA has poor solvent resistance, insufficient strength and low heat stability [19,20,21].

Meanwhile, among the conducting fillers, the water soluble and intrinsically conducting polymer poly(3,4-ethylenedioxythiophene):poly(styrenesulfonate) (PEDOT:PSS) can be utilized to engineer dielectric properties, owing to the unique dual-phase morphology of PEDOT:PSS. This consists of positively charged PEDOT-rich conducting domains dispersed within a negatively charged insulating PSS matrix and bonded through electrostatic force [20]. This enables PEDOT:PSS to simultaneously function as a conductive filler, ionic conductor, and interfacial modifier within polymer composite systems. The incorporation of PEDOT:PSS into polymer matrices introduces multiple polarization mechanisms that enhance the overall dielectric response due to the presence of -SO_3_H groups in the PSS chain that help in creating dipoles [6]. The ionic nature of the PSS counterion contributes to electrode polarization effects at low frequencies, while the electronic conductivity of PEDOT domains facilitates the formation of micro-capacitor networks, a characteristic of Maxwell Wagner Sillars (MWS) polarization [6]. The hydrophilic nature of PVA and PEDOT:PSS with -OH and -SO_3_H groups, respectively, makes it a sought-after combination for polymer dielectrics.

Another class of conducting fillers in trend is carbon nanomaterials like reduced graphene oxide (RGO). These materials have the exceptional properties of pristine graphene with enhanced processability and interfacial reactivity with an extraordinarily high aspect ratio [22]. This enables efficient load transfer by percolation network formation at low loading levels [23]. The presence of residual oxygen functional groups, including hydroxyl, epoxy, and carboxyl groups, facilitates strong interfacial interactions with polar polymer matrices while providing sites for chemical modification and functionalization. The electrical properties of RGO are governed by the complex interplay between the restored π-conjugated network and the disruption caused by residual defects and functional groups [23].

Many studies have been conducted on the role of PEDOT:PSS in PVA-based composites for enhancing the dielectric permittivity and AC conductivity of composites. Some examples are PVA/PEDOT:PSS, PVA/PEDOT:PSS/GO, PVA/PEDOT:PSS/MnO_2_ PVA/PEDOT:PSS/PPy/NiO, PVA/PVP/PEDOT:PSS/NiO and CMC/PVA/PEDOT:PSS/NiO nanoparticles [6,20,21,24,25,26]. The report on PVA/PEDOT:PSS/MnO_2_ composites showed composition- and temperature-dependent dielectric properties, with an optimized MnO_2_ concentration providing enhanced permittivity while controlling dielectric loss [20]. The report on complex PVA/PVP/PEDOT:PSS/NiO nanocomposites focused on how NiO concentration influenced the dielectric constant, dielectric loss and AC conductivity together with the structural and optical properties [26]. Similarly, NiO incorporation into CMC/PVA/PEDOT:PSS and PVA/PEDOT:PSS/PPy matrices enhanced dielectric polarization and electrical conductivity, demonstrating the important role of heterogeneous polymer–nanoparticle interfaces in controlling electrical response. However, these studies have largely focused on improving dielectric properties through metal-oxide loading, with investigations restricted to dielectric measurements near room temperature [21,25]. The role of RGO in various dielectric composites has been studied too, such as in situ-prepared PVA–RGO composites [22]. Here, the dielectric relaxation was attributed to interfacial effects arising from the formation of bound polymer layers at the RGO surface [22]. A remarkable dielectric enhancement (permittivity values exceeding 10^6^) was observed in PEDOT:PSS/expanded graphene/GO composites and was attributed to micro-capacitor formation and enhancement of MWS polarization. However, the work did not address the specific mechanisms governing filler–filler interactions or the role of the polymer matrix in mediating these interactions and their role in the relaxation process [27]. A report on a PEDOT:PSS–RGO@PU composite showed ultra-high permittivity (350 at 1 kHz), low dielectric loss (0.2 at 1 kHz), low loss shear modulus (200 MPa), and a low loss tangent (0.4). However, temperature-dependent dielectric properties were not measured [28]. Hebbar et al. (2017) [29] reported the influence of 1 and 5 wt.% GO and RGO nanoparticles loaded into a PVA–PEDOT:PSS blend. Again, the dielectric properties were attributed to MWS polarization and an increase in conductivity to percolation at room temperature, though temperature-dependent analysis was absent [29]. Similarly, another report by Hebbar et al. (2019) focused on free volume and conduction rather than detailed dielectric relaxation across temperature or frequency [30]. Another report on this peculiar combination of materials was reported by Yin et al. (2022), focusing on conductive fiber performance fabricated through electrospinning rather than temperature-dependent dielectric studies [31].

Previous studies have demonstrated that incorporation of graphene-derived fillers into PVA/PEDOT:PSS systems can modify electrical and dielectric properties. However, comparatively less attention has been given to the evolution of these effects over a broad temperature and frequency window and to distinguishing interfacial polarization from increasingly dominant charge transport contributions at elevated temperature. Accordingly, the present study focuses on the systematic influence of RGO concentration on the dielectric response of a PP30 matrix over 303–523 K and 10^2^–10^6^ Hz and correlates these changes with structural, vibrational, morphological and thermal characteristics of the composites.

## 2. Materials and Methods

### 2.1. Preparation of RGO–PEDOT:PSS–PVA Composites

The reduction of GO was carried out by a procedure reported elsewhere [32,33]. A GO suspension prepared by a modified Hummers method from pure graphite (Alfa Aesar 250 mesh (Alfa Aesar, Haverhill, MA, USA) was washed with diluted HCl (Merck (Darmstadt, Germany), MW = 36.46 g/mol, Assay = ≥35%, HCL) and double-distilled water until pH was 7. The resultant product was dried at 318 K for 24 h. The GO suspension obtained was exfoliated in water using ultrasonication for 1 h at 300 K. A total of 1 μL of hydrazine monohydrate (Sigma Aldrich (St. Louis, MO, USA), 64–65%, reagent grade, ≥97%) for 1 mg GO was added to GO in water suspension and stirred at 373 K for 24 h for reduction of GO. The RGO precipitate was separated and washed several times by methanol and double-distilled water and dried at 333 K [33].

**Figure 1:**
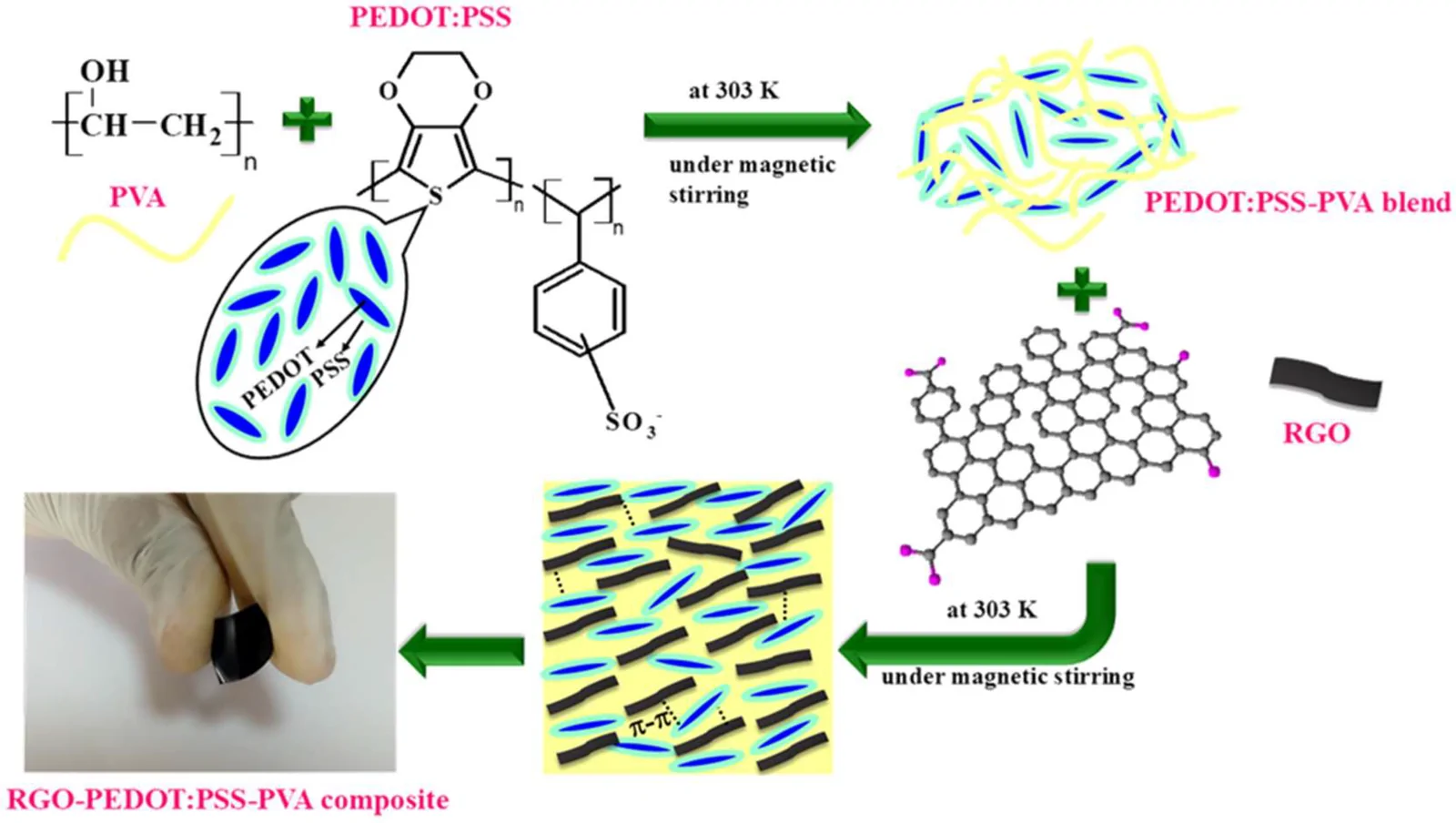
Schematic of formation of RGO–PEDOT:PSS–PVA composite and interaction between GO and PEDOT:PSS within PVA matrix.

The optimized concentration of the blend comprising 30 vol.% of PEDOT:PSS in PVA (PP30) was prepared by the same method reported in our previous work, where a 5 *w/v* % PVA solution was prepared in double-distilled water by adding PVA and stirring at 358 K for 2 h. The PVA was procured from Qualigens, Khalapur, India, that is 85–89% hydrolyzed, and as-purchased PEDOT:PSS from Sigma Aldrich (1.3 wt.% in H_2_O) was used for preparation of samples. The concentration of PEDOT:PSS in 10 mL of PVA varied from 10 to 50 vol.%; as-purchased PEDOT:PSS was blended at 300 K for 1 h under magnetic stirring. The solutions were cast at 300 K for 30 days in a petri dish to obtain freestanding films. Based on the best morphology and electrical properties analyzed through various characterization techniques, we chose 30 vol.% of PEDOT:PSS in PVA (PP30) for further studies [6]. RGO was dispersed in 1 mL of dimethyl sulfoxide (DMSO) (Sigma Aldrich, ACS reagent, ≥99.9%) to form 0.5, 1 and 1.5 mg/mL of RGO solution. The prepared dispersions were added into 10 mL of PP30 solution and stirred vigorously at 303 K for 1 h using a magnetic stirrer. The composites were formed by solvent casting at 303 K for two months. The composites with 0.5, 1 and 1.5 mg/mL RGO in PP30 were denoted as PPRGO0.5, PPRGO1 and PPRGO1.5 respectively. The interactions between PVA, PEDOT:PSS and RGO in RGO–PVA–PEDOT:PSS are represented schematically in Figure 1.

### 2.2. Characterization

The powder X-ray diffraction (XRD) pattern was recorded at a scanning rate of 0.02° by using Rigaku Ultima III with CuK_α1_ radiation of wavelength 1.5406 Å in the range of 10–80°. The Fourier transform infrared spectra for all the samples were collected in the range of 4000–500 cm^−1^ using a Thermo Scientific Nicolet IS5 spectrophotometer (Thermo Scientific, Waltham, MA, USA). For the thermal stability of the samples, thermogravimetric analysis (TGA) and their derivatives (DTG) were obtained by a Perkin Elmer STA6000 analyzer (Perkin Elmer, Springfield, IL, USA) in the range of 313–1023 K under the flow of N_2_ atmosphere. A microRaman analysis was performed for all the samples in the range of 50–4000 cm^−1^ using a LabRAM HR microRaman spectrophotometer (HORIBA Scientific, Kyoto, Japan) with an excitation wavelength of 532 nm. The morphology of the prepared freestanding films was captured using a Quanta 250 FEG (FEI, Hillsboro, OR, USA), FEI field emission scanning electron microscope (FE-SEM). The atomic force microscope (AFM) and a Kelvin probe microscope (KPFM) (Multimode 8, Bruker, Billerica, MA, USA) were taken to analyze the dispersion of RGO and confirm well exfoliated RGO nanosheets. The conductive microcantilever was used with a spring constant of 2 N m^−1^ and a resonance frequency of 70 kHz (OPUS, 240AC-PP). The fabricated films were of average thickness: 141 μm for PP30 and 300 μm for RGO-incorporated PP30 samples [Mitutoyo litematic VL-50 (Mitutoyo, Kawasaki, Japan) digimatic measuring unit (0.15 N force)]. A 1.5 × 1.5 cm^2^ portion was cut from the fabricated films, and conducting silver paste was coated on either side of the films to form a parallel plater capacitor. The frequency- and temperature-dependent dielectric measurements were made by using a Hioki 3532-50 LCR Hi-Tester (Hioki, Ueda, Japan) within the temperature range of 303–523 K for frequency 10^2^–10^6^ Hz. Analysis of dielectric data and modeling in the frequency domain was carried out using bespoke software, DATAMA (Datama_modernized_31052025), based on the software package MATLAB 2025b^©^ (Math Works Inc., Natick, MA, USA) [34].

## 3. Results and Discussion

### 3.1. Results

**Figure 2:**
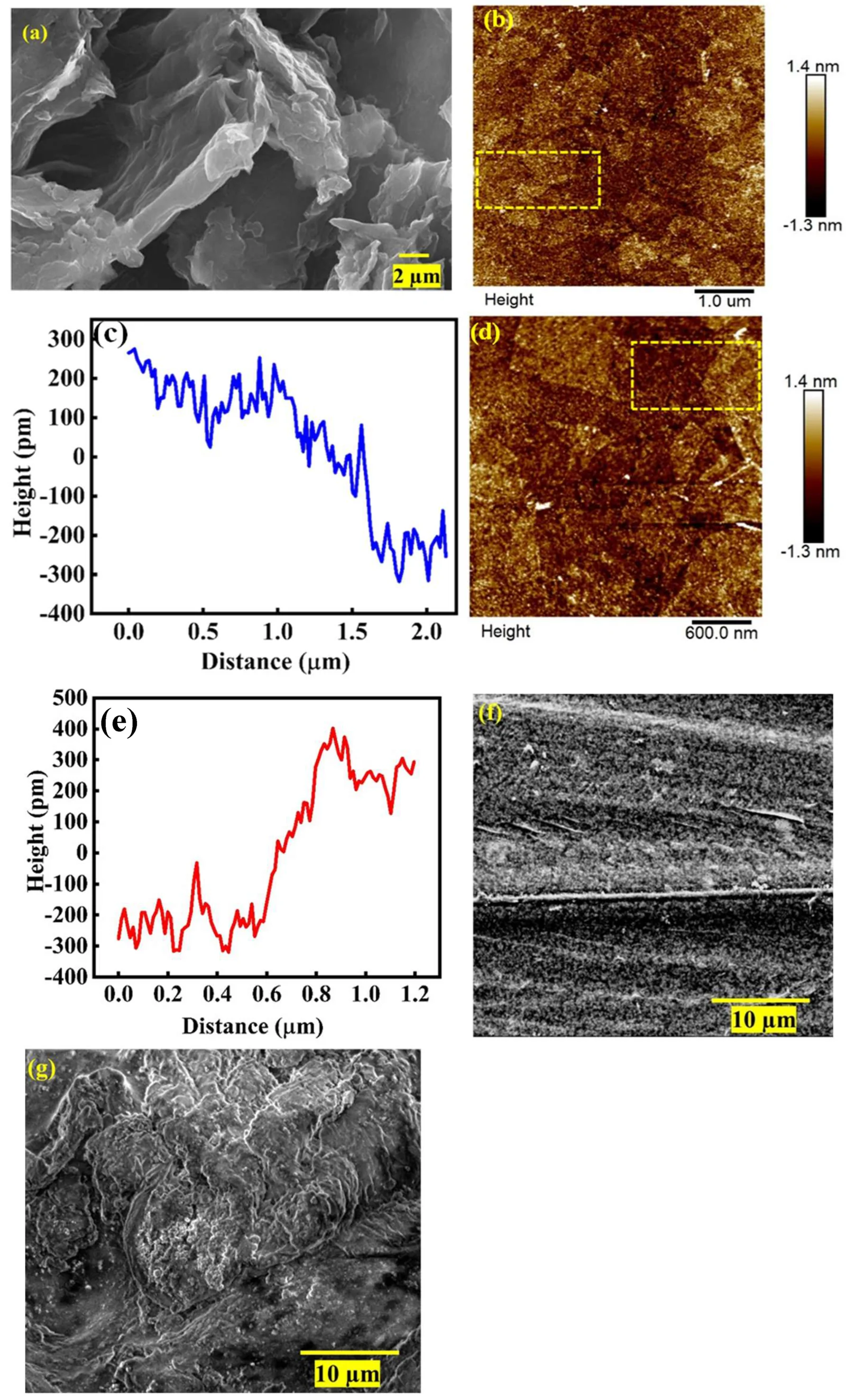
FE-SEM images of (**a**) RGO, AFM topographic images of (**b**–**e**) RGO in DMSO dispersion and (**c**) line profile of AFM image (**b**). (**e**) Line profile for AFM image and (**d**) FE-SEM images of (**f**) PP30 and (**g**) PPRGO1.

The morphological changes leading to the interaction between PVA, PEDOT:PSS and RGO depicted in Figure 1 are captured by the FE-SEM images in Figure 2. Figure 2a suggests a well exfoliated two-dimensional sheet of RGO, while PP30 in Figure 2f shows a packed structure of PEDOT:PSS embedded in the PVA matrix, where the darker or black regions correspond to the insulating PVA matrix, while the brighter ones correspond to the conducting PEDOT:PSS filler. Figure S1 in Supporting Information shows the FE-SEM of plain PVA, with only a dark region of polymer. Upon the addition of RGO in PP30(PPRGO1), the RGO layers seem to enwrap the PP30 within, with brighter clusters coming together in contact with RGO, suggesting the enhanced interaction between the primary filler PEDOT:PSS and secondary filler RGO within the PVA matrix as a result of the π–π interaction along with intermolecular hydrogen bonding, and can be observed in Figure 2g [24,27]. The AFM topographic imaging of RGO in Figure 2b,d revealed laterally extended, ultrathin sheet-like domains with wrinkled and localized regions of overlap. The 5 × 5 µm^2^ and 3 × 3 µm^2^ scans showed irregular height distributions rather than a uniformly flat surface, consistent with the characteristic folding or wrinkling of reduced graphene-based sheets. Representative line profiles in Figure 2c,e yielded local vertical height differences of approximately 0.55 and 0.62 nm, respectively, demonstrating sub-nanometer topographic variations across selected RGO domains. These values are consistent with the presence of very thin RGO sheets; however, because an exposed SiO_2_/Si substrate baseline could not be clearly identified along the selected profiles, the measured values are conservatively interpreted as relative step-height differences rather than absolute monolayer thicknesses. The coexistence of extended low-height regions and locally elevated domains further suggests partial exfoliation accompanied by localized sheet overlap/restacking [35].

**Figure 3:**
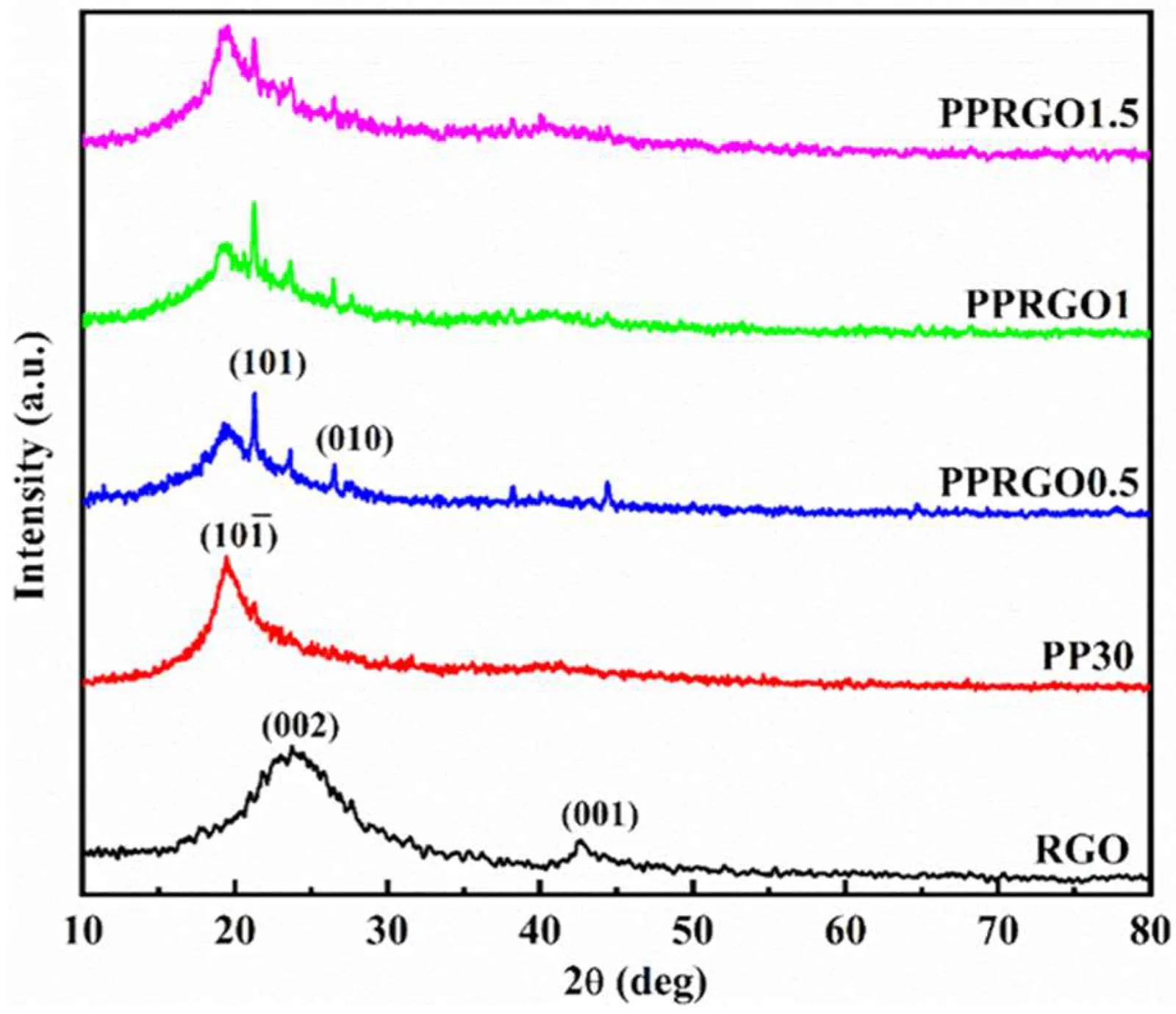
Powder XRD patterns for samples RGO, PP30, PPRGO0.5, PPRGO1 and PPRGO1.5, respectively.

The powder XRD patterns for RGO, PP30, PPRGO0.5, PPRGO1 and PPRGO1.5 are shown in Figure 3. The characteristic XRD peak pattern for RGO is in line with the previous literature reports [36,37]. The semicrystalline peak at 19.44° in PP30 corresponded to the Bragg’s reflection from (10$\bar{1}$) plane of PVA, with a value of d = 4.6 Å, owing to the atactic PVA trans planar conformation, while the broad hump at 40.52° (d = 2.2 Å) corresponds to hexagonal ordering of molecular chains in PVA. The absence of any additional peaks corresponding to PEDOT:PSS indicated a strong dissolution of PEDOT:PSS within the PVA matrix [6,38]. The decrease in intensity of the peaks at 19.44° and 40.52° for PPRGO0.5 and PPRGO1 suggests a reduction in the crystallinity of samples with the addition of RGO. Moreover, minor peak position variations of 21.3°, 21.24° and 21.24° for PPRGO0.5, PPRGO1 and PPRGO1.5, respectively, were observed for these composites but cannot be accounted as substantial changes to account for any structural changes. RGO-incorporated samples showed the reappearance of a dormant peak corresponding to (101) and (010) of the PVA backbone with the addition of RGO.

**Figure 4:**
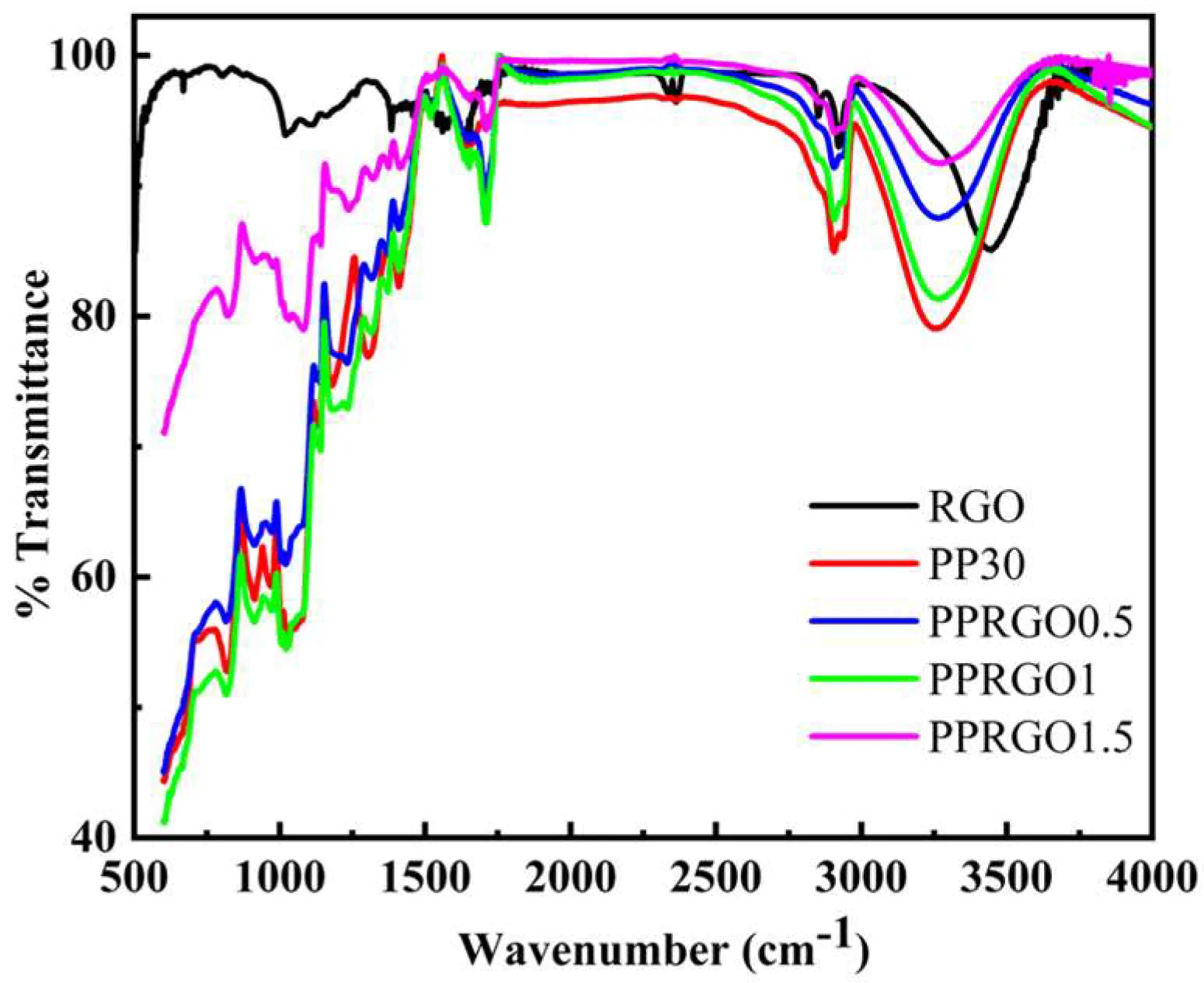
FTIR spectra for samples RGO, PP30, PPRGO0.5, PPRGO1 and PPRGO1.5.

The FT-IR spectra for the RGO–PEDOT:PSS–PVA composites are depicted in Figure 4. All the characteristic vibrational bands observed for the pristine RGO, PP30, PPRGO 0.5, PPRGO1 and PPRGO1.5 samples are given in Table S1 of supporting information. The characteristic vibrational bands corresponding to RGO were consistent with the literature reports [36,39]. Most of the vibrational bands corresponding to PP30 are due to the skeletal PVA matrix along with the presence of additional vibrational bands corresponding to PEDOT:PSS, indicating the successful complexation of PEDOT:PSS with PVA through intermolecular hydrogen bonding between the sulfonate groups of PSS and hydroxyl groups in the PVA [6,40,41,42,43,44,45]. However, the characteristic bands for ν (C-O-C) and ν (C-O) of the PVA matrix were absent for PP30. The addition of RGO shows a shift in the band position of PP30 (tabulated in Table S1).

**Figure 5:**
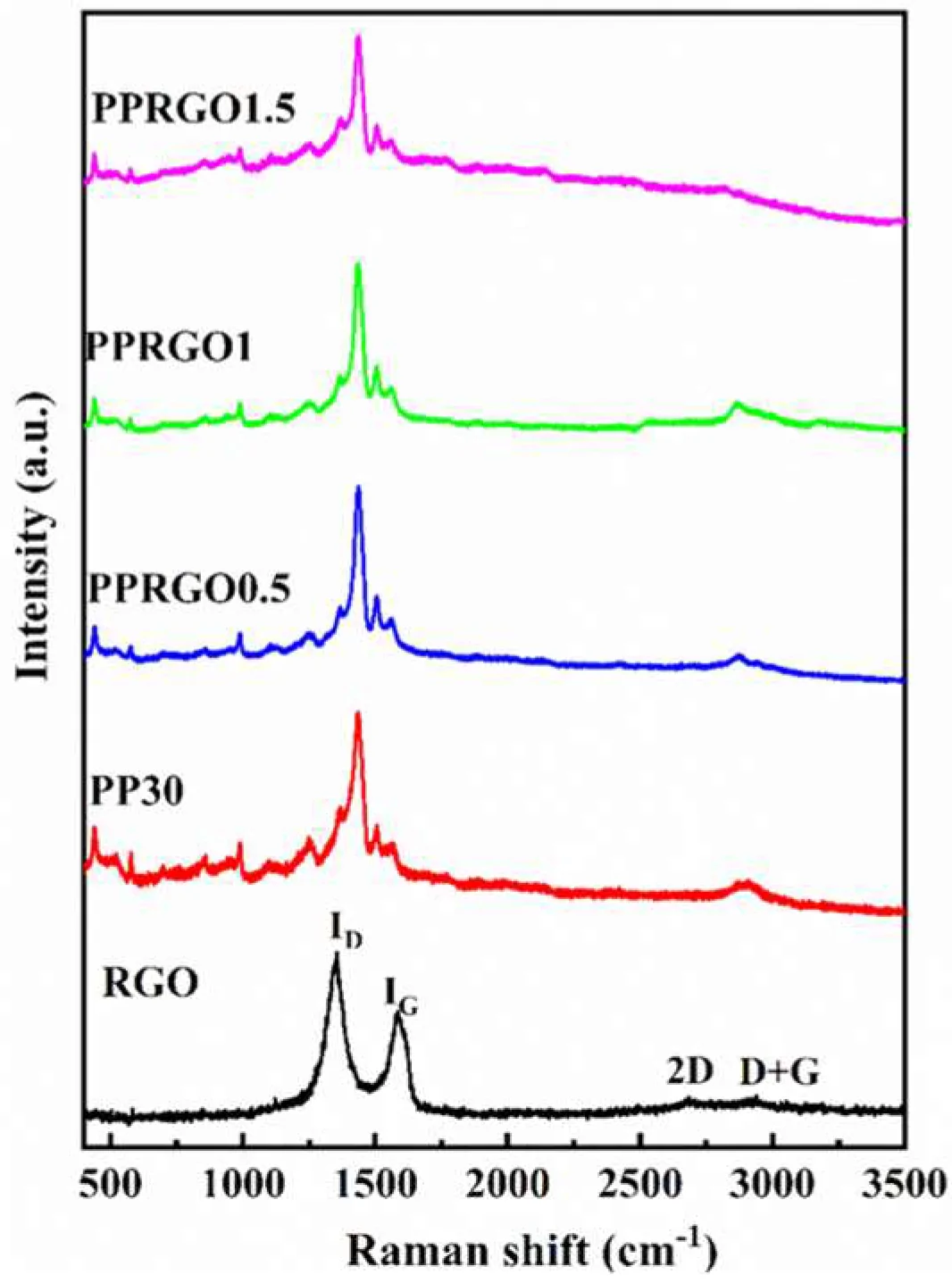
MicroRaman spectra for samples RGO, PP30, PPRGO0.5, PPRGO1 and PPRGO1.5, respectively.

In support of FT-IR data, microRaman spectra for RGO–PEDOT:PSS–PVA composites are provided in Figure 5. The D and G vibrational bands of RGO were recorded at 1349 and 1582 cm^−1^, respectively. The additional bands of 2D at 2681 cm^−1^ and D + G at 2886 cm^−1^ arise from the defects present in the graphitic structure of the carbon material upon reduction, indicative of better graphitization of RGO [36]. The microRaman spectrum for PP30 consists of typical Raman fingerprints of PEDOT, PSS and PVA. The scattering peaks 2904 and 2872 cm^−1^ suggest stretching vibration of -CH_2_ of PVA. The prominent vibrational modes observed for the RGO–PEDOT:PSS–PVA composites are summarized in Table S2 in Supporting Information [46,47].

**Figure 6:**
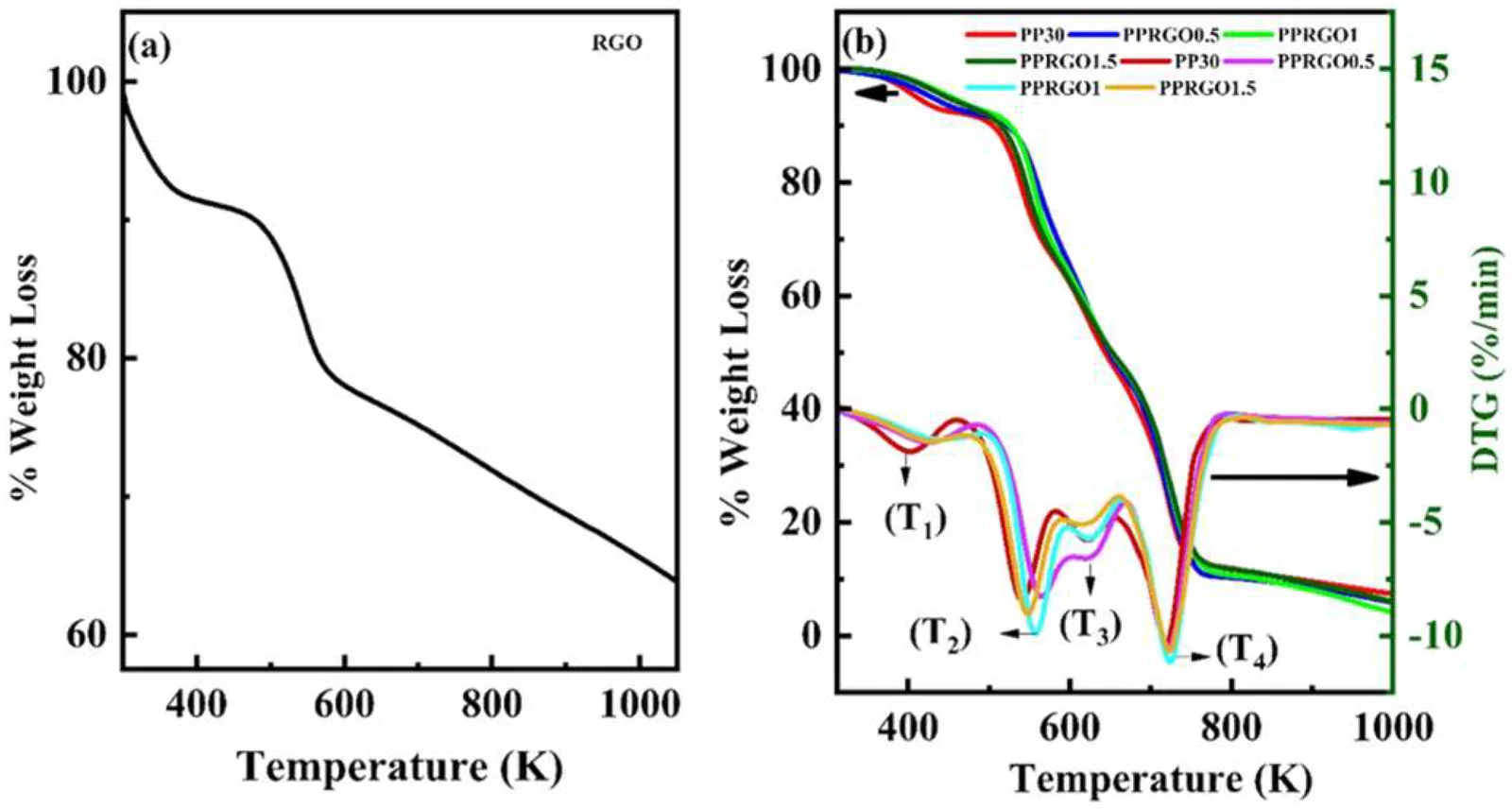
(**a**) TGA of RGO and (**b**) TGA and DTG plots for samples PP30, PPRGO0.5, PPRGO1 and PPRGO1.5.

The thermal stability of the RGO–PEDOT:PSS–PVA composite was determined using the TGA and DTG plots depicted in Figure 6. RGO underwent a two-step degradation, as in Figure 6a, while samples PP30, PPRGO0.5, PPRGO1 and PPRGO1.5 underwent a four-step degradation, as seen in Figure 6b. The two-step degradation in RGO was observed, with the first step around 300–420 K, with a weight loss of 8.86%, due to the desorption of physically adsorbed moisture and/or residual volatile species, consistent with the hydrophilic nature of PVA and PSS in the PP30 matrix. Meanwhile, the second step of degradation was observed in the range of 420–603 K, with a further weight loss of 13.2%, due to the decomposition of less stable oxides or oxygen functional groups in the form of H_2_O, CO_2_, and CO gases. The slow rate of weight loss observed above 603.7 K is attributed to the removal of a more stable oxide such as carboxyl groups [48]. A total of 34.4% weight loss at 1000 K is observed for RGO. The four-step thermal decomposition in Figure 6b for all the samples is ascribed to the PVA and PEDOT:PSS backbone of the composite, where step I is contributed by the removal of residual water molecules, step II and III are due to the combined effect of the breaking up of the side chains of PVA and the oxidizing decomposition of the skeletal PEDOT and/or PSS backbone chain structures, while step IV is due to decomposition of the main chain of the PVA matrix or carbonization of residual polymer segments [20,49]. With the addition of RGO, the changes in weight loss percentage for each step along with the maximum peak temperatures in DTG were noted and are recorded in Table S3 in supporting information.

### 3.2. Discussion

The entanglement of RGO sheets within PP30, as seen in Figure 2c, resulted in breaking of intermolecular hydrogen bonding within the PVA chain. It also enhanced electrostatic interaction between RGO and PEDOT:PSS sheets, causing the XRD peaks in Figure 3, with a change in intensity of the peak at 19.44°, minor peak position variations though not substantial, and the emergence of a new peak. However, the possibility of recrystallization of the PVA/PEDOT:PSS matrix by RGO, changing the intermolecular distance of the polymer backbone, an increase in intensity for the peak corresponding to (10$\bar{1}$) plane of PVA in PPRGO1.5 [50]. The presence of new peaks at 26.52°, 26.44° and 26.5° for PPRGO0.5, PPRGO1 and PPRGO1.5, respectively, corresponds to interchain π–π stacking along (010) plane of the thiophene ring [51]. The new peaks at 23.62°, 23.66° and 23.66°, corresponding to PPRGO0.5, PPRGO1 and PPRGO1.5, respectively, correspond to (002) plane of RGO in the composites [37]. The peaks observed in the RGO-incorporated samples in the ~23–27° and ~44° regions are consistent with contributions from RGO-derived graphitic ordering and modified short-range ordering within the polymer composite, as per the literature reports. Because the differences in peak position are small, these shifts cannot be used to qualitatively suggest evidence of changes in π–π stacking distance. The relatively stronger polymer-related scattering observed for PPRGO1.5 may indicate partial recovery of chain ordering at the highest RGO loading and may possibly be associated with increased RGO–RGO association and reduced disruption of the polymer matrix. The turbostratic band of RGO appears for samples PPRGO0.5, PPRGO1 and PPRGO1.5 at 44.4°, 44.44° and 44.44°, indicative of short-range order in stacked RGO layers.

This rearrangement in overall π-conjugation of the system with addition of RGO into PP30 is supported by FTIR data in Table S1. The addition of RGO resulted in the reduction in intensity as well as a blue shift in the band position of the ν(-OH) band, suggesting the active participation of hydroxyl groups of PVA in intermolecular hydrogen bonding with functional groups in RGO as well as PEDOT:PSS through PSS groups. The hydrogen bonding helped in the segregation of PSS from PEDOT, with PEDOT being exposed to RGO through π–π interaction [6]. Additionally, the red shift and increase in intensity of the band corresponding to 1651 cm^−1^ for PP30 with the addition of RGO are attributed predominantly to the bending vibration of absorbed/bound water associated with the hydrophilic PVA matrix, with a possible contribution from residual oxygen-containing groups of RGO [40,52]. However, the apparent return of the band corresponding to 1651 cm^−1^ for PPRGO1.5 reflects a change in the local hydrogen-bonding/water environment and band overlap. This may be possibly associated with partial RGO restacking and a lower effective polymer–RGO interfacial area. The ν(C=C) peak, corresponding to thiophene ring stretching, was shifted to 1519, 1520 and 1522 cm^−1^ for PPRGO0.5, PPRGO1 and PPRGO1.5, respectively. The intensity of the peak increased with RGO content, except for PPRGO1.5, which decreased. The addition of RGO resulted in the appearance of a strong new band corresponding to ν(-C=O) at 1709, 1708 and 1780 cm^−1^ for PPRGO0.5, PPRGO1 and PPRGO1.5, respectively [36]. Also, a blue shift was observed for bands corresponding to ν(-C-S) stretching, an indicator of the better intercalation of RGO into the PP30 matrix because of rearrangement of the PEDOT:PSS structure in PP30 with the incorporation of RGO, evidenced from the shift in the stretching vibrational bands of the −${\mathrm{SO}}_{3}^{-}$ group in PSS. Further, a new band corresponding to −${\mathrm{SO}}_{3}^{-}$ asymmetric stretching was observed at 1231, 1234 and 1237 cm^−1^ for PPRGO0.5, PPRGO1 and PPRGO1.5, respectively [51].

**Figure 7:**
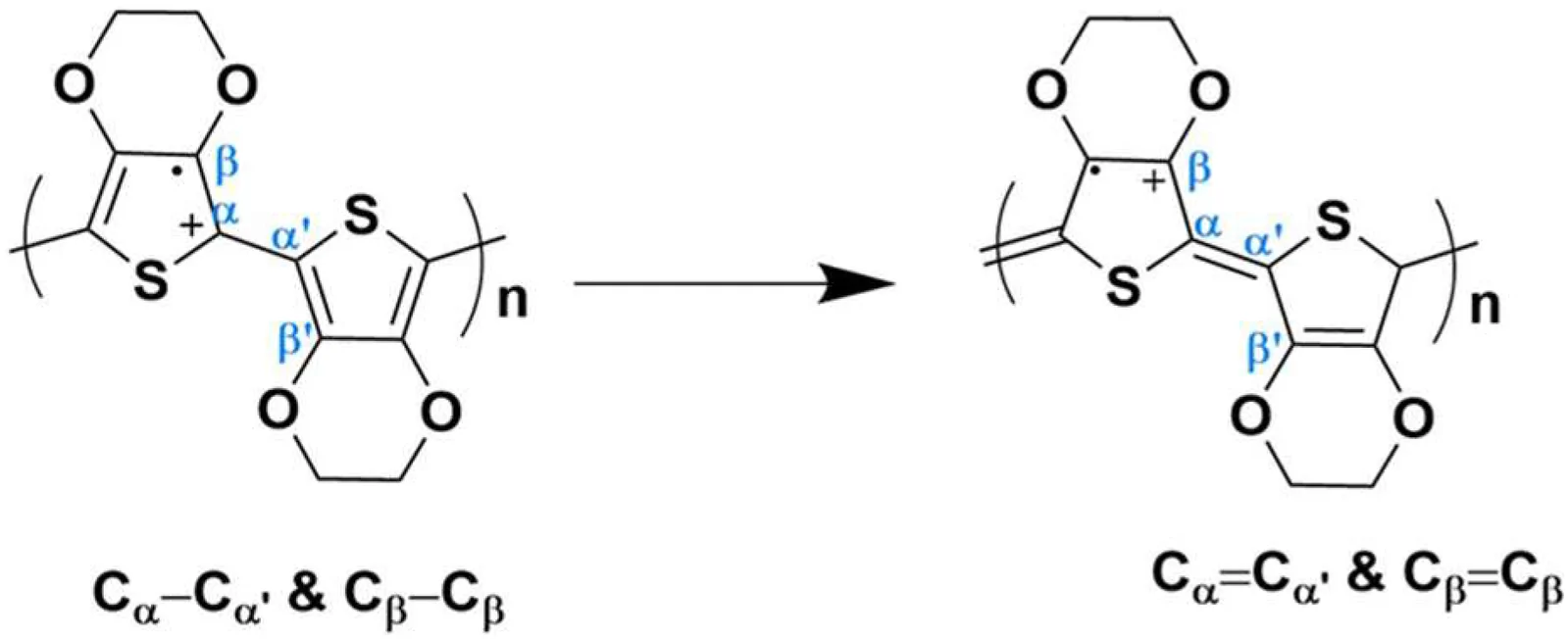
Benzoid-to-quinoid transition due to C_β_-C_β_ to C_β_=C_β_ and C_α_-C_α′_ to C_α_=C_α′,_ transformation.

The microRaman data in Figure 5 and Table S2 supports the claim of a change in π-conjugation, where shifting Cα=C_β_ band position indicates a change in π-electron cloud along the planar PEDOT ring, altering the conjugation length [53]. The increment in the intensity of the Raman band corresponding to PEDOT for C_β_-C_β_ and C_α_-C_α′_ stretching along with a minor red shift in the bands for C_β_-C_β_ along with C_α_-C_α′_ shifting indicates a transformation from C_β_-C_β_ to C_β_=C_β_ and C_α_-C_α′_ to C_α_=C_α′,_ respectively, and is depicted in Figure 7 [47]. These changes suggest a structural rearrangement of PEDOT:PSS, most likely the benzoid-to-quinoid transition, exposing more PEDOT moieties to RGO and resulting in enhanced π-stacking. However, PPRGO1.5 underwent no change in the band positions for C_β_-C_β_ and C_α_-C_αʹ_ stretching. The band representing oxyethylene ring deformation at 860 cm^−1^ suggests the change in structural alignment of the PEDOT moiety in the composite upon addition of RGO [54]. Similarly, the band corresponding to PSS at 1097 cm^−1^ underwent a blue shift, while the PSS band at 1562 cm^−1^ red shifted, indicating weakened electrostatic interaction between sulfonic acid groups of PSS and PEDOT upon the interaction of RGO. The addition of RGO to PP30 resulted in enhanced thermal stability of the composite, as evidenced by the shift in DTG peaks in Figure 6b. The sample PPRGO1 was found to exhibit better thermal stability, with DTG peak maxima T1, T2, T3 and T4 at 441.49, 557.16, 625.95 and 723.67 K, respectively, owing to rearrangement of π-electron cloud, as evidenced by FTIR and microRaman data, and the tight enwrapping of RGO sheets with PEDOT:PSS in PP30 in FE-SEM images. The breaking of intermolecular hydrogen bonding and structural rearrangement caused T1 and T2 to change from 400.58 and 441.49 K for PP30 to 541.55 and 557.16 K, respectively. The intensities of the DTG peaks T1 and T3 are significantly reduced with content of RGO, suggesting that RGO acts as a thermal barrier and heat-dissipating filler, providing a better operating temperature window for use in practical application high-temperature polymer capacitors.

**Figure 8:**
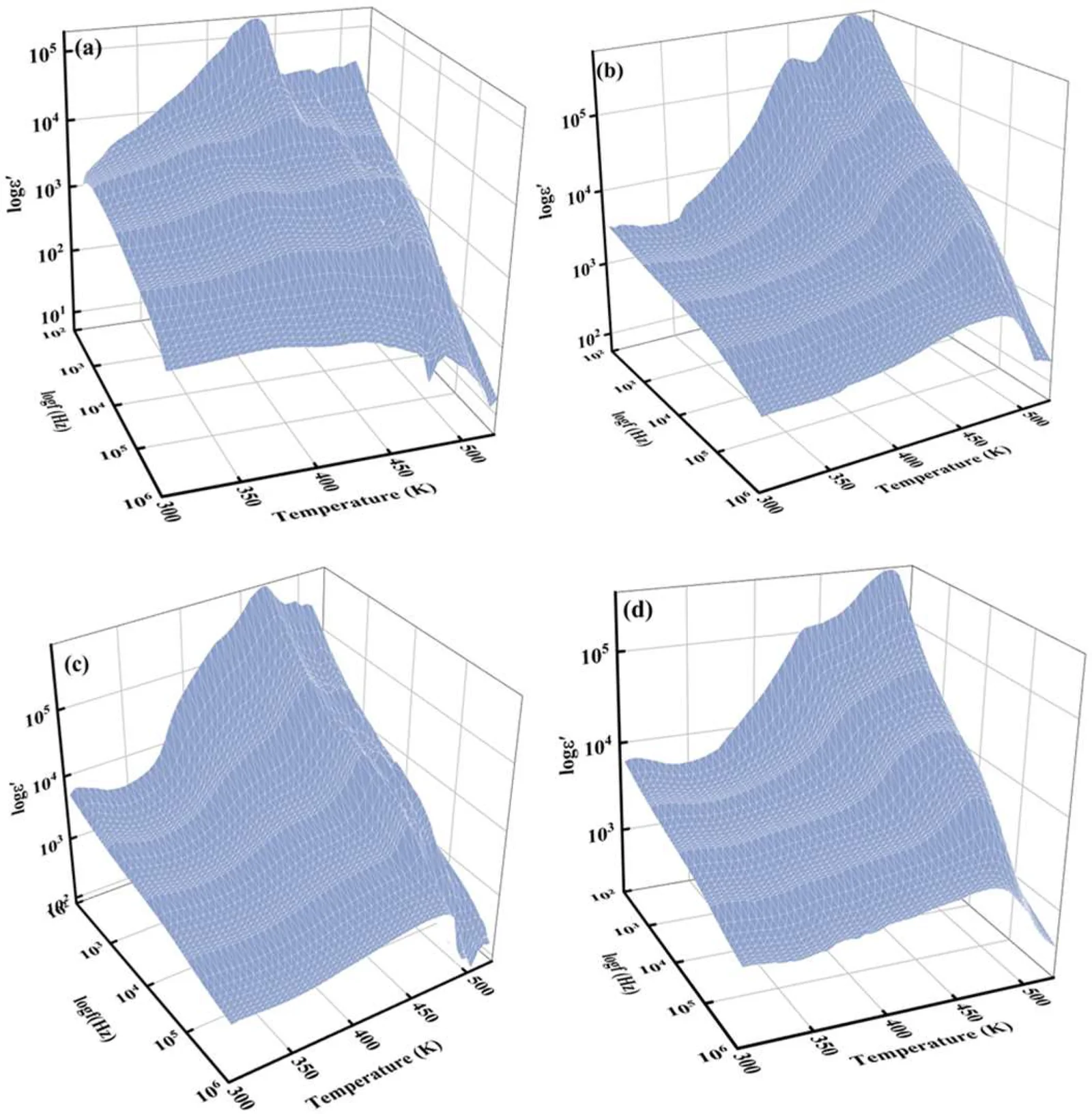
3D plots of real part of permittivity *ε′* at various temperatures from 303 to 523 K for samples (**a**) PP30, (**b**) PPRGO0.5, (**c**) PPRGO1 and (**d**) PPRGO1.5, respectively.

**Figure 9:**
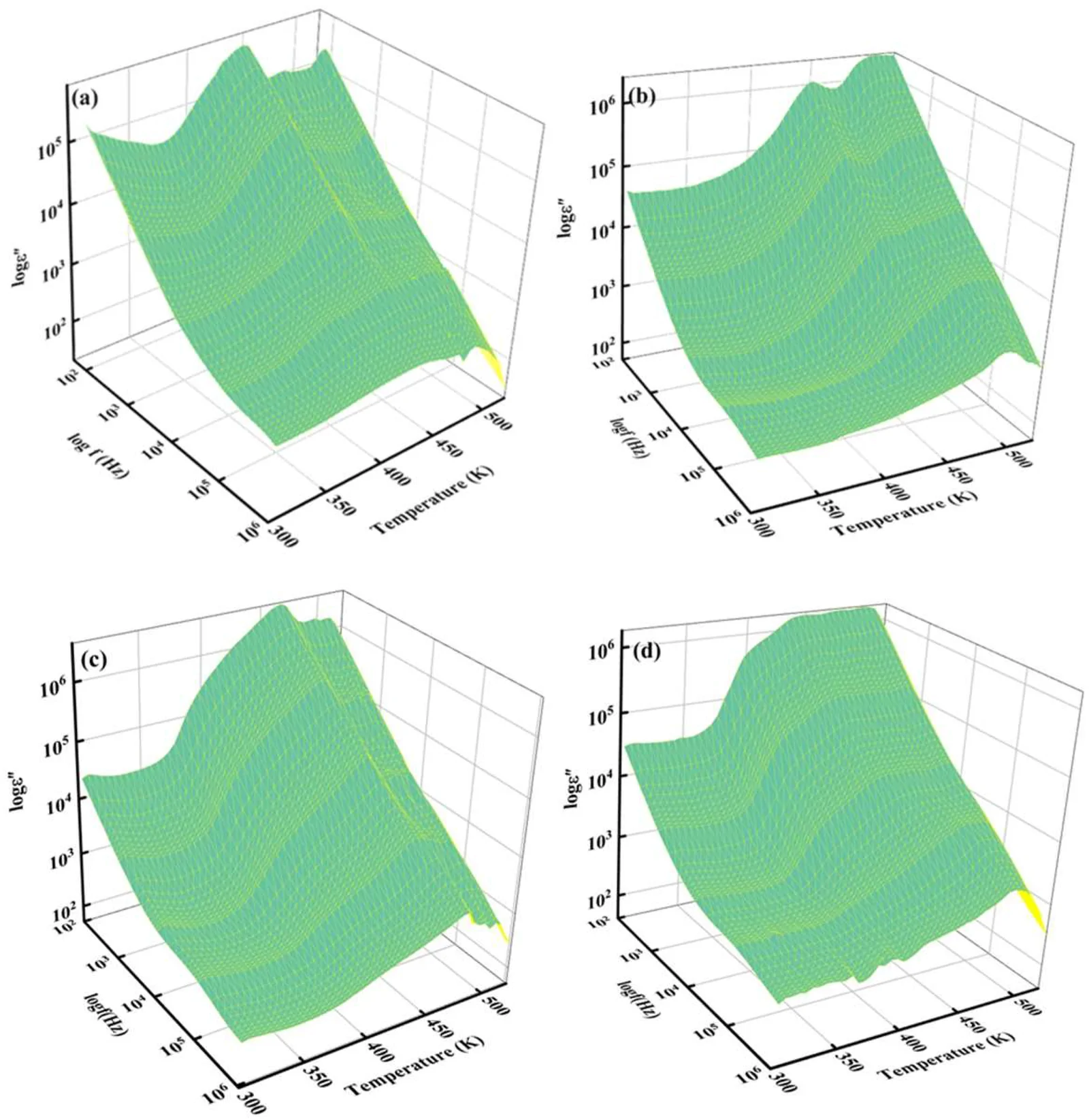
3D plots of imaginary part of permittivity *ε″* at various temperatures from 303 to 523 K for samples (**a**) PP30, (**b**) PPRGO0.5, (**c**) PPRGO1 and (**d**) PPRGO1.5, respectively.

The 3D plot for real part of permittivity *ε′* plotted against frequency range 10^2^ to 10^6^ Hz and temperature range 303–523 K for the PP30, the base matrix and its RGO-filled composites, namely PPRGO0.5, PPRGO1, and PPRGO1.5, is presented in Figure 8. As shown in Figure 8, the real permittivity *ε′* increases with both rising RGO content and temperature, whereas the *ε′* decreases with increasing frequency. This is a clear indication of interfacial polarization at low frequency, and as the frequency increases, the interfacial charges cannot follow the rapidly oscillating field, and their contribution diminishes, leading to the observed reduction in ε′. The addition of RGO significantly modifies the dielectric response of the PP30 matrix. At 303 K and 100 Hz, the dielectric constant increases from approximately 3.2 × 10^3^ for PP30 to 4.9 × 10^3^ for PPRGO1 and 6.1 × 10^3^ for PPRGO1.5. The enhancement of the value of *ε′* at the lower frequency regime is owed to Maxwell Wagner Sillars (MWS) polarization or interfacial polarization, where charge carriers accumulate at the boundaries between the highly conductive RGO sheets and the complexed, relatively insulating PEDOT:PSS–PVA matrix [20,29]. MWS polarization is supported by the FT-IR and Raman spectroscopy data confirming the successful primary complexation of PEDOT:PSS with PVA via intermolecular hydrogen bonding, which provides matrix stability. Crucially, the introduction of RGO is confirmed to facilitate strong π–π interactions between the π-electron rich graphitic domains of RGO and the conductive PEDOT chains, as suggested by shifts in the Raman bands for the composite. The FE-SEM images (Figure 2) visually support this, showing RGO layers effectively enwrapping the PP30 clusters and acting as numerous micro-capacitor networks within the bulk material. These specific, low-resistance, molecular-level π–π interfaces maximize the trapping and accumulation of charge carriers under an applied electric field, thereby explaining the increase in macroscopic value of *ε′*. As the temperature increases from 303 K to 523 K, ε′ increases substantially in the low-frequency region for all samples. The increase arises from enhanced mobility of dipolar segments in the polymer chains and thermally activated charge carriers that accumulate at internal interfaces. At around 423 K, ε′ reaches values approaching 10^5^, indicating a strong contribution from space-charge polarization [29]. However, the influence of RGO loading is not purely monotonic. Although PPRGO1.5 shows the highest permittivity at room temperature, the PPRGO1 composite exhibits the most stable dielectric response across the frequency range, particularly at intermediate temperatures. This suggests that, at approximately 1 *w*/*v*. % RGO, the graphene sheets are sufficiently dispersed to maximize interfacial polarization without forming extended conductive pathways. In contrast, extremely large ε′ values observed for PPRGO0.5 at 523 K are accompanied by large dielectric losses, indicating that conduction-related polarization begins to dominate at elevated temperatures, as in Figure 9.

Figure 9 illustrates the 3D plot of *ε*″ against frequency ranging from 10^2^ to 10^6^ Hz and temperature range from 303 to 523 K, which represents energy dissipation or dielectric loss. At 303 K, PP30 shows very high dielectric loss in the low-frequency region, with ε″ reaching approximately 2.7 × 10^5^ at 100 Hz. Such high loss is typical of conducting polymer systems where mobile charge carriers contribute to dielectric dissipation and the dc component of conductivity, *σ_dc_*, begins to dominate. The addition of RGO significantly reduces dielectric loss at room temperature. For example, ε″ decreases to approximately 4.0 × 10^4^ in PPRGO0.5 and further to 2.2 × 10^4^ in PPRGO1. This reduction indicates that graphene sheets modify the internal charge transport pathways within the composite. When well dispersed, graphene can promote interfacial polarization while simultaneously restricting long-range carrier migration, thereby reducing dielectric dissipation [29]. As the temperature increases, dielectric loss increases for all samples, particularly at low frequencies. At 423 K, ε″ increases by nearly an order of magnitude relative to its value at 303 K. This increase indicates thermally activated charge transport processes, where the increase in ε″ with temperature is associated with enhanced hopping conduction of charge carriers [55]. Very large ε″ values appear in the low-frequency region, especially for PPRGO0.5. This behavior indicates the increasing contribution of conductive losses and electrode polarization at elevated temperatures. However, PPRGO1 and PPRGO1.5 maintain comparatively lower dielectric loss at higher frequencies.

**Figure 10:**
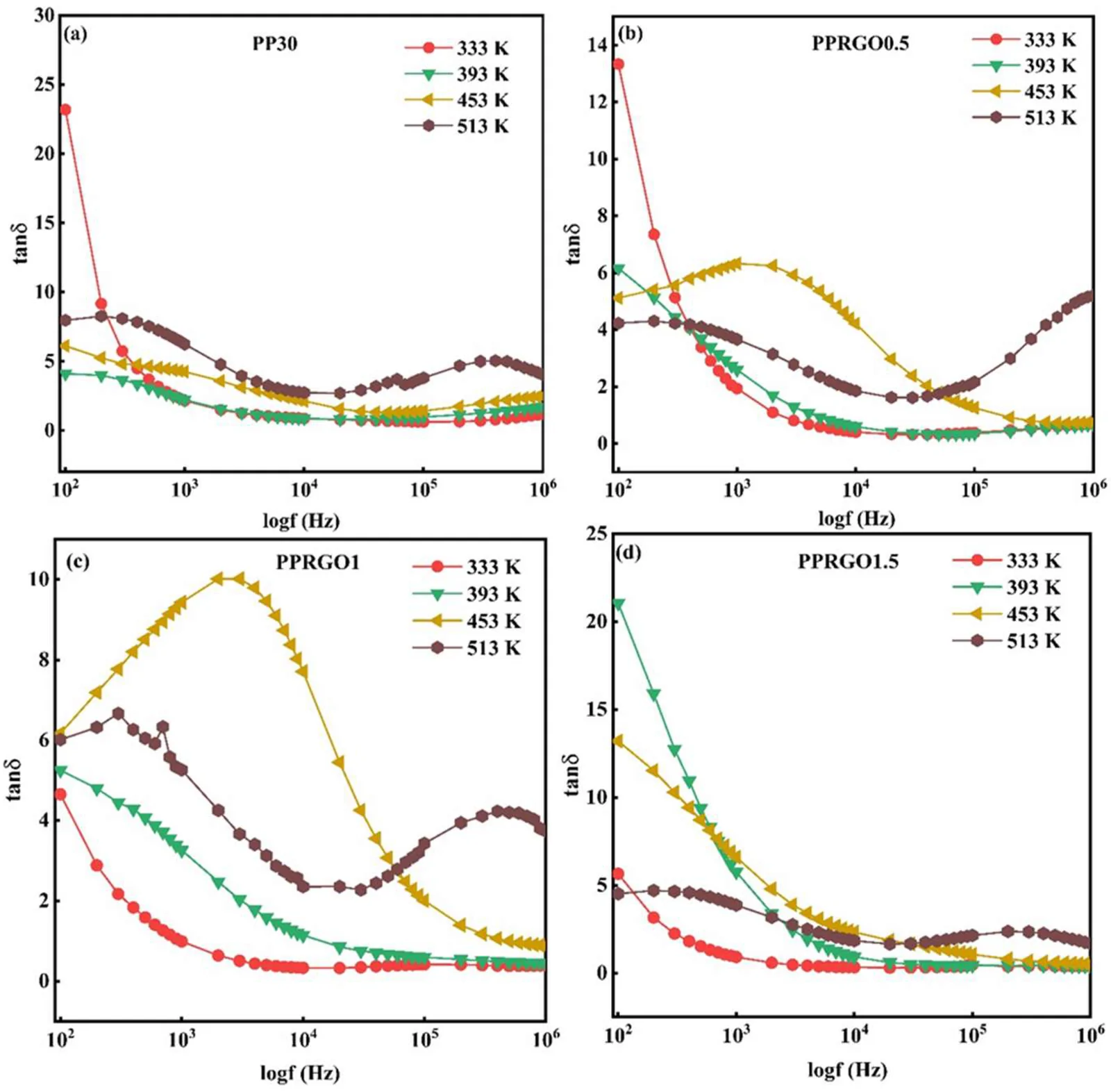
Frequency-dependent tan δ plots for samples (**a**) PP30, (**b**) PPRGO0.5, (**c**) PPRGO1 and (**d**) PPRGO1.5 at selected temperatures of 333, 393, 453 and 513 K respectively.

The loss tangent, tan δ, spectra in Figure 10 show that dielectric dissipation is controlled by both RGO concentration and temperature. At 333 K, the pronounced decrease in tan δ with increasing frequency indicates that energy dissipation is dominated by slow charge motion at low frequencies, whereas these charges cannot follow the rapidly oscillating field at higher frequencies [56]. The marked reduction in tan δ from 23.18 for PP30 to 4.65 for PPRGO1 at 333 K and 100 Hz suggests that an intermediate RGO loading restricts long-range charge transport through redistribution and partial trapping of carriers at RGO–polymer interfaces [57]. With increasing temperature, thermally activated carrier mobility becomes prominent, and the loss spectra broadens. PPRGO1.5 exhibits particularly high low-frequency loss at 393–453 K, whereas PPRGO1 develops a broad kHz-range loss maximum at 453 K, showing that interfacial polarization and charge transport increasingly overlap [58]. At 513 K, all samples display broad minimum, followed by renewed high-frequency loss, indicating a complex superposition of distributed relaxation and conductive contributions. Overall, RGO does not simply reduce dielectric loss; rather, it controls the balance between interfacial polarization and carrier transport, with PPRGO1 providing better results.

**Figure 11:**
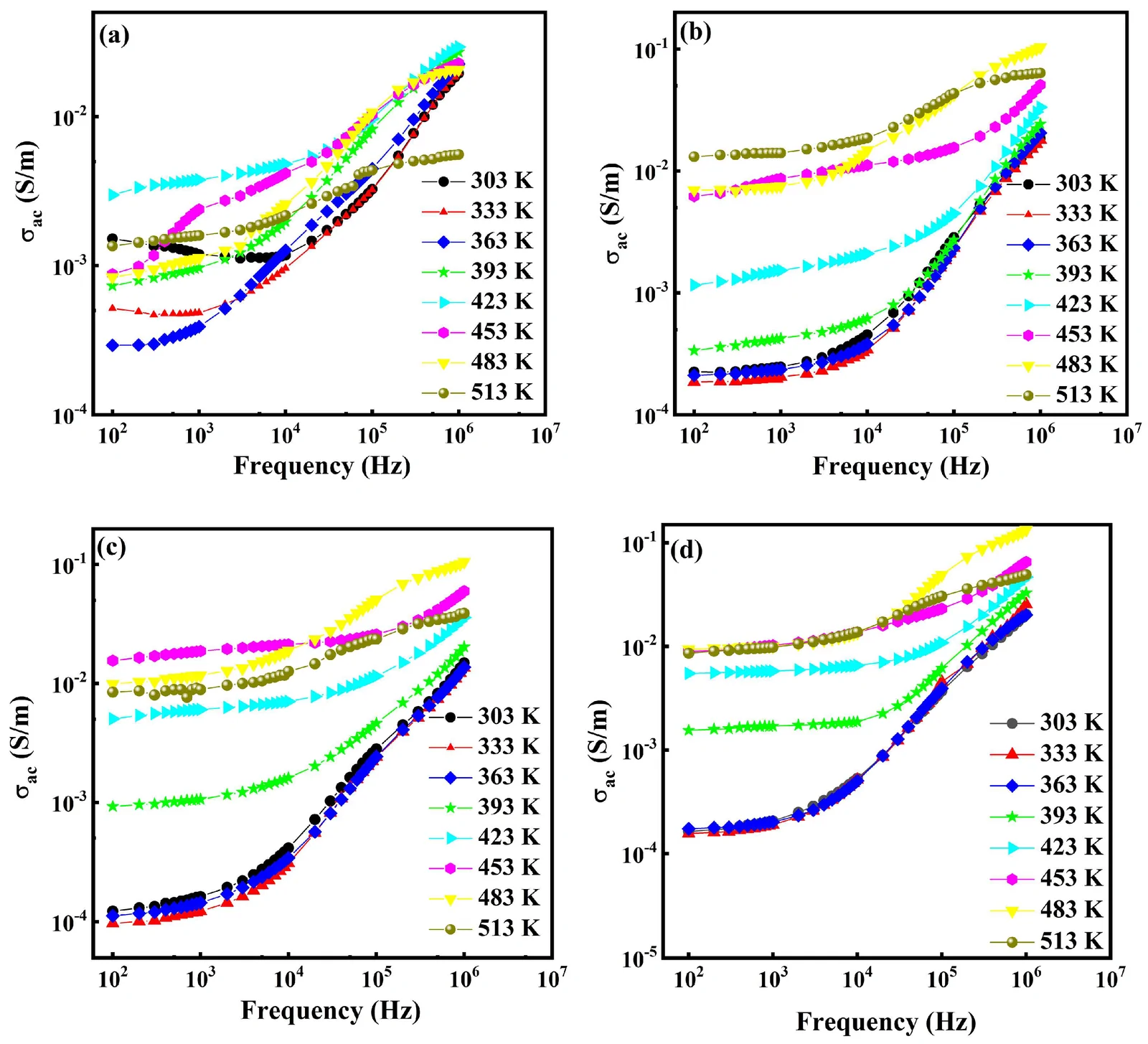
Frequency dependence of σ_ac_ for (**a**) PP30, (**b**) PPRGO0.5, (**c**) PPRGO1 and (**d**) PPRGO1.5 at various temperatures.

The *σ_ac_* as a function of frequency for the PP30, PPRGO0.5, PPRGO1 and PPRGO1.5 samples at various temperatures is depicted in Figure 11 and follows Jonscher’s power law given by Equation (1) [55]:

*real*(*σ_ac_*) = *σ_dc_* + *Aω^s^* (1)

where the presence of two distinct regimes, a low-frequency plateau corresponding to DC conductivity (*σ_dc_*) and a high-frequency dispersion (*Aω^s^*), confirms the transition from long-range conduction to localized short-range motions, *s* is a temperature and frequency-dependent exponent with a value of 0 ≤ *s* ≤ 1, *ω* = 2πf, and *A* is a temperature-dependent component that influences the degree of polarizability. For all samples, *σ_ac_* exhibits a weak frequency-dependent region at low frequencies, followed by a dispersive increase at higher frequencies. At 303 K, the low-frequency conductivity at 100 Hz is 1.52 × 10^−3^ Sm^−1^ for PP30, while there are lower values of *σ_ac_* of about 2.25 × 10^−4^, 1.23 × 10^−4^ and 1.64 × 10^−4^ Sm^−1^ for PPRGO0.5, PPRGO1 and PPRGO1.5, respectively. This indicates that, at room temperature, charge transport is limited by interfacial barriers between the polymer matrix and graphene sheets. However, toward the high-frequency region (10^6^ Hz), the *σ_ac_* increases to 1.95 × 10^−2^, 1.85 × 10^−2^, 1.48 × 10^−2^ and 2 × 10^−2^ Sm^−1^ for PP30, PPRGO0.5, PPRGO1 and PPRGO1.5, respectively, suggesting enhanced localized hopping through graphene-assisted conductive domains [27,59].

With increasing temperature, *σ_ac_* increases significantly for the RGO-filled composites due to thermally activated carrier hopping. At 453 K and 100 Hz, conductivity increases to 6.19 × 10^−3^ Sm^−1^ (PPRGO0.5), 1.56 × 10^−2^ Sm^−1^ (PPRGO1), and 8.9 × 10^−3^ Sm^−1^ (PPRGO1.5) compared with 8.79 × 10^−4^ Sm^−1^ for PP30, indicating that graphene facilitates charge transport within the PEDOT:PSS network. The highest conductivity occurs around 483 K, where *σ_ac_* at ~10^6^ Hz reaches ~1.0 × 10^−1^ Sm^−1^ for PPRGO1 and PPRGO1.5, significantly higher than ~2.0 × 10^−2^ Sm^−1^ for PP30. This enhancement can be attributed to improved carrier mobility and reduced hopping distance provided by the interconnected PEDOT:PSS–RGO conductive pathways. At 513 K, *σ_ac_* slightly decreases in the high-frequency region, indicating saturation of thermally activated transport and possible structural rearrangement of the conductive network.

**Figure 12:**
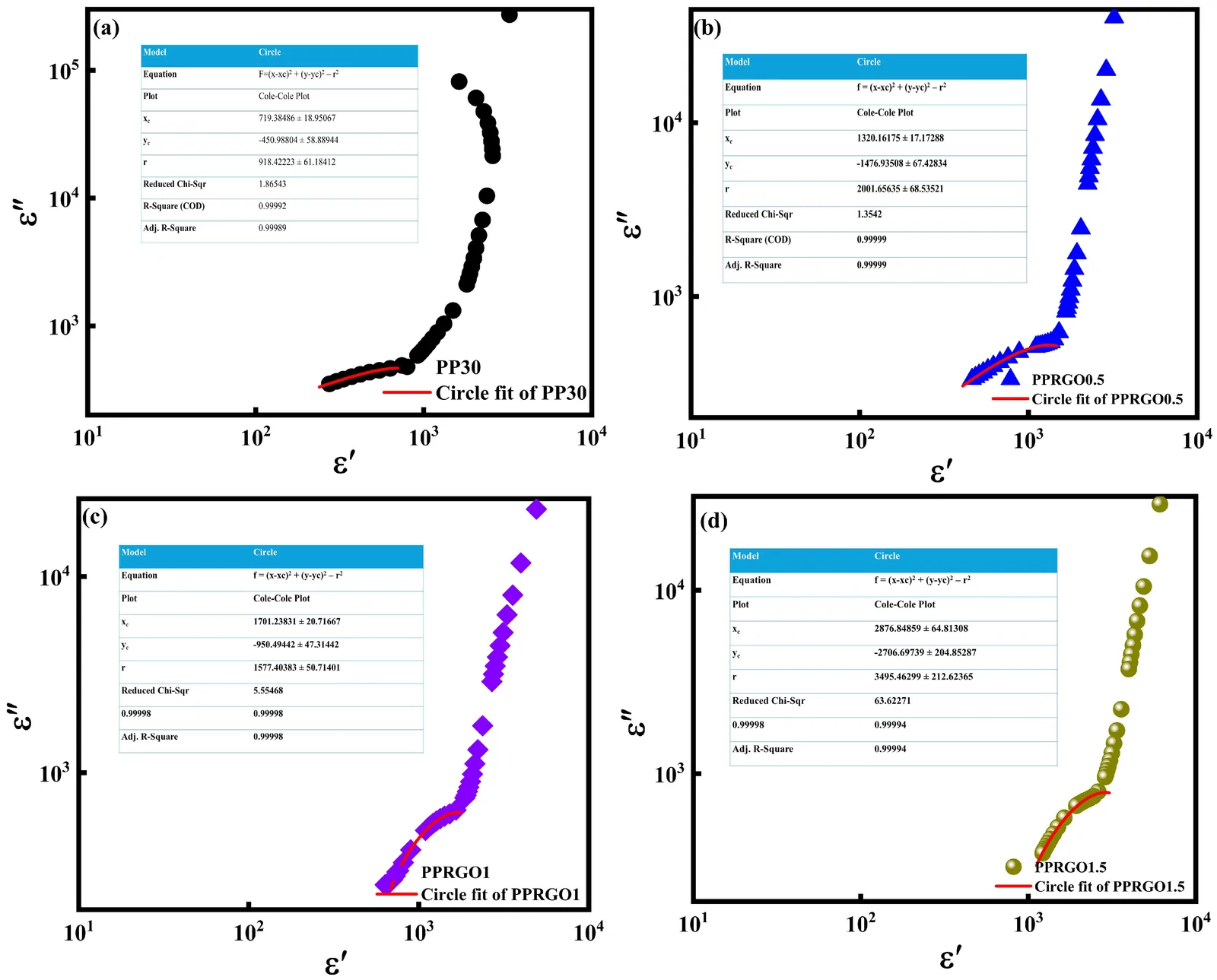
Cole–Cole Plot (ε′ vs. ε″) for (**a**) PP30, (**b**) PPRGO0.5, (**c**) PPRGO1 and (**d**) PPRGO1.5 at 303 K.

The Cole–Cole plots of PP30, PPRGO0.5, PPRGO1 and PPRGO1.5 at 303 K are shown in Figure 12. The deviation from an ideal semicircle, indicating non-Debye dielectric relaxation, was observed for all samples. This type of non-Debye relaxation has also been previously reported for PVA/RGO composites [22]. The nearly semicircular part of each experimental curve was fitted using

(2) $${\left(\varepsilon^{\prime }-x_{c}\right)}^{2}+{\left(\varepsilon \prime \prime -y_{c}\right)}^{2}=R^{2}$$

where *x_c_* and *y_c_* are the coordinates of the circle center for *ε′* and *ε″*, respectively, and *R* is the radius. The fitted curves agree closely with the selected experimental regions, with R^2^ (COD) values > 0.999 for PP30, PPRGO0.5, PPRGO1 and PPRGO1.5, respectively, as shown in the inset table in Figure 11. The Cole–Cole parameter, *α*_CC_, calculated from the depression of the fitted circle,

(3) $$\alpha_{\mathrm{CC}}=\frac{2}{\pi }\sin^{-1}\left(\frac{|y_{c}|}{R}\right)$$

was 0.327 for PP30, 0.528 for PPRGO0.5, 0.412 for PPRGO1 and 0.564 for PPRGO1.5. Here, *α*_CC_ = 0 represents ideal Debye relaxation, while increasing *α*_CC_ indicates a broader distribution of relaxation times [60,61].

*α*_HN_ = 1 − *α*_CC_ (4)

where *α*_HN_ is the corresponding Havriliak–Negamic (HN)-form broadening factors, which would be approximately 0.673, 0.472, 0.588 and 0.436, while *β* = 1 is fixed rather than experimentally extracted from the circle geometry.

Among the samples, PP30 has the lowest *α*_CC_ value of 0.327, indicating the relatively narrowest distribution of relaxation times. Addition of RGO increases the relaxation broadening, but the change is non-monotonic with RGO concentration. PPRGO0.5 shows a larger value of *α*_CC_ of 0.528, whereas PPRGO1 decreases to 0.412. Thus, among the RGO-containing samples, PPRGO1 shows the comparatively narrower distribution of relaxation times. This suggests a more uniform effective dielectric environment at this intermediate RGO concentration. At the highest RGO content, PPRGO1.5 shows the largest *α*_CC_ value (0.564), indicating a broad distribution of relaxation times. This behavior is consistent with increased dielectric heterogeneity at higher RGO loading, where polymer–RGO interfaces and local RGO–RGO proximity can provide different environments for polarization and charge motion. Figure 12 shows the strong upward deviation or tail in the experimental data from the fitted circles toward the low-frequency region. This shows that a single Cole–Cole relaxation cannot describe the complete dielectric response. The low-frequency deviations from the fitted arcs further indicate that dielectric relaxation occurs together with charge-related polarization and conduction processes contribute to *ε″*. Therefore, the fitted *α*_CC_ values describe mainly the relaxation-dominated part of the measured response, rather than the entire frequency range. This interpretation is consistent with previous studies of PVA/RGO composites, where dielectric relaxation was found to be non-Debye and strongly influenced by the heterogeneous polymer/filler environment and charge-carrier dynamics. Overall, the Cole–Cole analysis shows that RGO changes the distribution of relaxation times in the PVA/PEDOT:PSS matrix rather than simply increasing or decreasing the relaxation strength.

**Figure 13:**
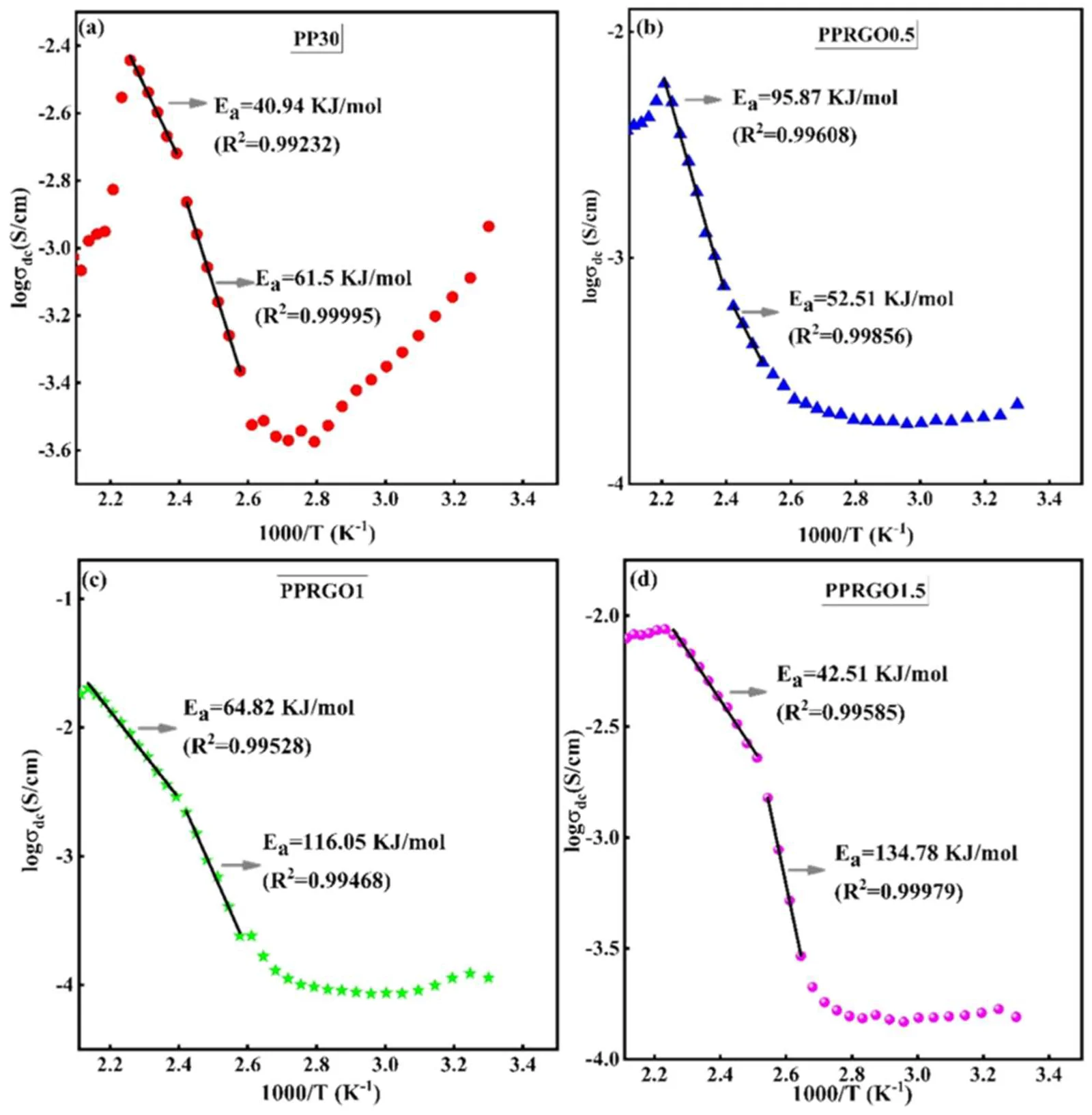
Plots for logσ_dc_ versus 1000/T (K^−1^) using Havriliak–Negami model for (**a**) PP30, (**b**) PPRGO0.5, (**c**) PPRGO1 and (**d**) PPRGO1.5 respectively.

The temperature dependence of *logσ_dc_* extracted from the general Havriliak–Negami model fitted using DATAMA [34], against inverse of temperature (1000/T), is shown in Figure 13 using a standard HN with conductivity form:

(5) $$A\varepsilon^{*}\left(\omega \right)=\varepsilon_{\infty }+\;\sum_{k}\frac{{∆\varepsilon }_{k}}{{\left[1+{\left(\mathrm{i\omega}\tau_{k\;}\right)}^{\alpha_{k}}\right]}^{\beta_{k}}}-i\frac{\sigma_{\mathrm{dc}}}{\varepsilon_{0}\omega^{s}}$$

where *ε*_∞_ is the high-frequency limiting permittivity; ∆*ε_k_* is the dielectric strength of relaxation *k*; *τ_k_* is its characteristic relaxation time; *α_k_* and *β_k_* describe the breadth and asymmetry of the relaxation-time distribution, respectively; *σ*_dc_ is the dc conductivity; *ε*_0_ is the vacuum permittivity; *ω* = 2*πf*, and *s* describes the frequency dependence of the conductivity contribution [34,62,63].

The conductivity increases with increasing temperature for all the samples, indicating thermally activated charge transport. The activation energy (*E_a_*) values obtained from the linear regions in Figure 13 represent the energy required for charge carriers to overcome interfacial barriers and participate in long-range electrical transport. The relatively lower activation energies observed for PP30 (40.94 and 61.50 kJ mol^−1^) indicate moderate thermally activated hopping within the PVA/PEDOT:PSS matrix. The value of conductivity at infinite temperature *σ*_0_ for PP30 obtained from the fitting result is 2.39 and 4.91 Scm^−1^, corresponding to *E_a_* values of 40.94 and 61.50 kJ mol^−1^ respectively. Upon the addition of RGO, the *σ*_0_ and *E_a_* values increase in high-temperature regions, especially in PPRGO1 (5.58 and 12.03 Scm^−1^, corresponding to 64.82 and 116.05 KJ mol^−1^, respectively) and PPRGO1.5 (2.95 and 15.09 Scm^−1^, corresponding to 42.51 and 14.78 KJ mol^−1^, respectively), indicating the formation of stronger interfacial trapping sites due to enhanced interaction between PEDOT:PSS and RGO sheets as supported by FTIR in Figure 4 and micro Raman data in Figure 5. At lower temperatures, charge carriers remain trapped within the PVA/PEDOT:PSS matrix because of restricted polymer chain mobility and interfacial barriers between PVA, PEDOT:PSS and RGO. As the temperature increases, the polymer segments gain sufficient mobility, and the trapped carriers are thermally activated, resulting in hopping conduction through interconnected PEDOT:PSS and RGO conductive domains. Therefore, the HN-derived *σ_dc_* results confirm that the incorporation of RGO modifies both polarization and conduction behavior through interfacial charge accumulation, hopping conduction and formation of localized conductive networks. Overall, the results demonstrate that RGO incorporation enhances thermally activated AC conduction, with PPRGO1 and PPRGO1.5 showing the most efficient carrier transport, particularly in the high-frequency region.

## 4. Conclusions

In summary, we successfully fabricated freestanding films of RGO–PEDOT:PSS–PVA composites via a cost-effective solution blending method, demonstrating a significant enhancement in both dielectric constant and AC conductivity through strategic incorporation of RGO. The key finding is the profound structural–electrical coupling achieved in the optimal PPRGO1 composite. At this loading, the composite exhibited a sharp increase in real permittivity, attributed to the dominant MWS interfacial polarization. The thermal stability of the material is improved synergy between PEDOT:PSS and RGO through π–π interaction, suggesting the prepared samples are highly promising material for applications requiring high charge storage capacity and improved conductivity, such as flexible electronics and advanced energy storage devices, with an improved operating temperature window.

## Data Availability

The original contributions presented in this study are included in the article/Supplementary Material. Further inquiries can be directed to the corresponding author.

## Supplementary Materials

The following supporting information can be downloaded at: https://www.mdpi.com/article/10.3390/polym18192403/s1. Figure S1: FESEM image of Pure PVA freestanding film; Table S1: FT-IR band assignments for RGO, PP30, PPRGO0.5, PPRGO1 and PPRGO1.5 respectively; Table S2: Prominent Raman shift observed for RGO-PEDOT:PSS-PVA composites; Table S3: Measured weight loss in percentage for samples, PP30, PPRGO0.5, PPRGO1 and PPRGO1.5 from TGA and peak maxima from DTG.
