# Supplementary material for: Dissecting the Role of Reduced Graphene Oxide on the Dielectric Properties of Poly(vinyl alcohol)/Poly(3,4-ethylenedioxythiophene):Poly(styrenesulfonate) Blend
Source: Polymers (Basel). 2026 Oct 1;18(19):2403. doi: 10.3390/polym18192403 (PMC13667233; doi:10.3390/polym18192403)
Supplement: Supplementary file 1 [file polymers-18-02403-s001.zip › polymers-4420048-supplementary.pdf]

<u>**SUPPORTING INFORMATION**</u>

Dissecting the Role of Reduced Graphene Oxide on the Dielectric Properties of Poly(vinyl alcohol)/Poly(3,4-ethylenedioxythiophene):Poly(styrenesulfonate) Blend

Mydhili Varma [1,2], Sellaperumal Manivannan [2] and Paul Ben Ishai [1,*]

[1] The Dielectric & THz Science Laboratory, Department of Physics, Ariel University of Samaria, Ariel 4070000, Israel; mydhili@ariel.ac.il

[2] Carbon Nanomaterials Laboratory, Department of Physics, National Institute of Technology, Tiruchirappalli 620 015, India; ksmani@nitt.edu

[*] Correspondence: paulbi@ariel.ac.il

FIGURES

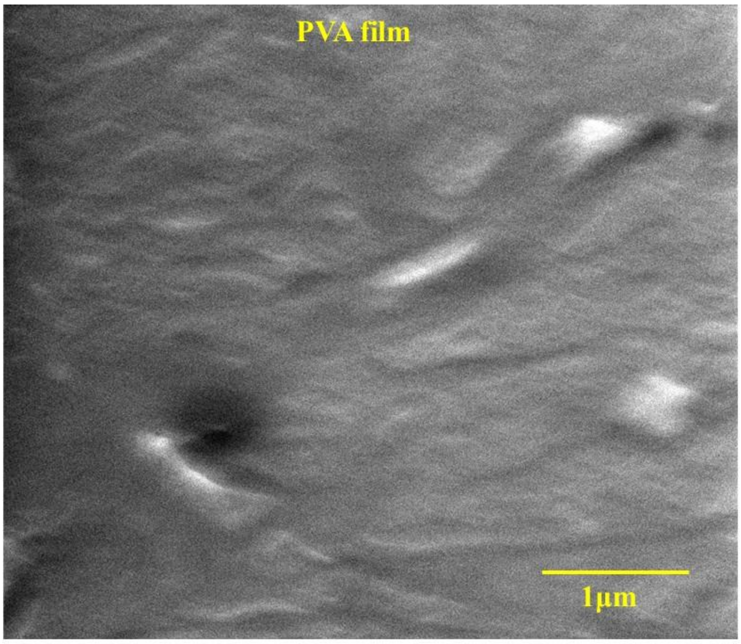

Figure S1. FESEM image of Pure PVA freestanding film.

The image displays gray scale as it is a nonconducting polymer and the strains show folds and creases in PVA sample that are normal for a peeled of freestanding film from substrate or mould after casting and is supported by reference 7 mentioned in the main manuscript.

TABLES

Functional groups	Wave number (cm^{-1})				
	RGO	PP30	PPRGO0.5	PPRGO1	PPRGO1.5
ν(-OH)	3441	3256	3262	3262	3268
ν_{as}(-CH_2)	2922	2936	2936	2935	2937
ν_s(-CH_2)	2852	2905	2905	2906	2907
ν(C=O)	1741	-	1709	1708	1780
ν(C=C) of unoxidized graphitic domains	1628	-	-	-	-
δ(H–O–H) of PVA	-	1651	1647	1644	1651
ν(-C=C) in thiophene ring of PEDOT:PSS	-	1519	1519	1520	1522
scissoring mode of -CH_2 in PVA	-	1408	1408	1408	1414
-CH_2 wagging and deformation in PVA	-	1372	1372	1372	1374
ρ(CH_2) in PVA	-	1303	1316	1316	1321
-SO_3^- asymmetric stretching in PSS	-	-	1231	1234	1237
Stretching of the ethylenedioxy thiophene ring in PEDOT	-	1178	1178	1176	1176
Symmetrical stretching vibrations of SO_3H in PSS	-	1139<br>1124	1139<br>1124	1139<br>1125	1140<br>1124
Stretching of -SO_3^- group in PSS	-	1078<br>1031<br>1005	1078<br>1020<br>1005	1078<br>1020<br>1005	1079<br>1024<br>1007
ν(C-O) of epoxy group	1020	-	-	-	-
ν(-C-S) stretching in PEDOT:PSS	-	969	972	973	977
ν(C-C) of PVA	-	913	913	913	916
C-H out of plane vibration of PVA	-	818	816	815	821

Table S1. FT-IR band assignments for RGO, PP30, PPRGO0.5, PPRGO1 and PPRGO1.5 respectively.

Vibrational band	Raman Shift (cm^{-1})				
	RGO	PP30	PPRGO0.5	PPRGO1	PPRGO1.5
I	1349				
G	1582				
2D	2681				
D+G	2886				
Asymmetric stretching of -CH_2 of PVA		2904	2979	2996	3001
Symmetric stretching of -CH_2 of PVA		2872	2872	2866	2822
Asymmetrical $C\alpha=C_\beta$ of thiophene ring		1504	1505	1505	1506
Symmetrical $C\alpha=C_\beta$ of thiophene ring		1434	1436	1434	1436
C_β-C_β stretching of thiophene ring		1368	1368	1366	1368
C_α-$C_{\alpha'}$ inter-ring stretching of thiophene ring		1250	1250	1251	1251
Vibrational mode of PSS		1562<br>1097<br>951	1560<br>1104<br>951	1559<br>1104<br>942	1559<br>1104<br>947
oxyethylene ring deformation		860	857	856	852

Table S2. Prominent Raman shift observed for RGO-PEDOT:PSS-PVA composites.

Sample Name	I (315-470.5 K)		II (470.5-570 K)		III (570-640.2 K)		IV (640.2-796.2 K)	
	Weight loss (%)	T_1 (K)	Weight loss (%)	T_2 (K)	Weight loss (%)	T_3 (K)	Weight loss (%)	T_4 (K)
PP30	7.86	400.58	23.42	541.55	17.84	623.01	38.87	720.98
PPRGO0.5	7.18	422.07	17.22	563.94	22.89	623.64	42.16	721.22
PPRGO1	6.05	441.49	22.09	557.16	19.07	625.95	42.02	723.67
PPRGO1.5	6.34	430.34	23.94	548.13	17.12	621.17	40.79	724.08

Table S3. Measured weight loss in percentage for samples, PP30, PPRGO0.5, PPRGO1 and PPRGO1.5 from TGA and peak maxima from DTG.
